# High charge-carrier mobility enables exploitation of carrier multiplication in quantum-dot films

**DOI:** 10.1038/ncomms3360

**Published:** 2013-08-23

**Authors:** C. S. Suchand Sandeep, Sybren ten Cate, Juleon M. Schins, Tom J. Savenije, Yao Liu, Matt Law, Sachin Kinge, Arjan J. Houtepen, Laurens D. A. Siebbeles

**Affiliations:** 1 Optoelectronic Materials section, Department of Chemical Engineering, Delft University of Technology, Julianalaan 136, 2628BL Delft, The Netherlands; 2 Department of Chemistry, University of California, Irvine, Irvine, California 92697, USA; 3 Toyota Europe, Materials Research & Development, Hoge Wei 33, B-1930 Zaventem, Belgium

## Abstract

Carrier multiplication, the generation of multiple electron–hole pairs by a single photon, is of great interest for solar cells as it may enhance their photocurrent. This process has been shown to occur efficiently in colloidal quantum dots, however, harvesting of the generated multiple charges has proved difficult. Here we show that by tuning the charge-carrier mobility in quantum-dot films, carrier multiplication can be optimized and may show an efficiency as high as in colloidal dispersion. Our results are explained quantitatively by the competition between dissociation of multiple electron–hole pairs and Auger recombination. Above a mobility of ~1 cm^2^ V^−1^ s^−1^, all charges escape Auger recombination and are quantitatively converted to free charges, offering the prospect of cheap quantum-dot solar cells with efficiencies in excess of the Shockley–Queisser limit. In addition, we show that the threshold energy for carrier multiplication is reduced to twice the band gap of the quantum dots.

## Abstract (web summary)

Carrier multiplication effects are of promise for enhancement of solar cells, but have been difficult to exploit in such devices. Here, the authors demonstrate how carrier multiplication in quantum-dot films can be considerably enhanced by appropriate tuning of the charge-carrier mobility.

## Graphical abstract

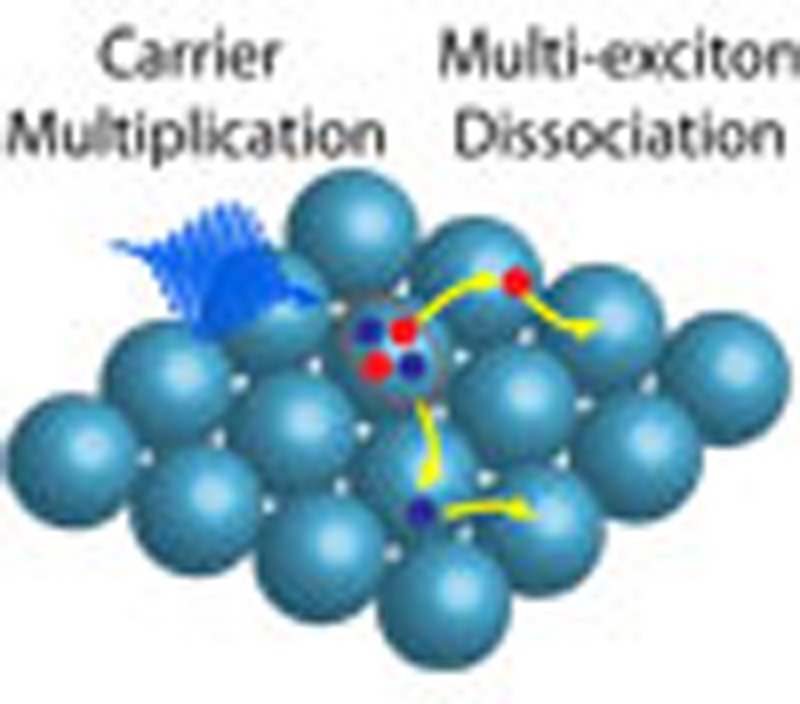

The generation of multiple electron–hole (e–h) pairs by the absorption of a single photon, usually termed carrier multiplication (CM) or multiple exciton generation, has been a topic of intense research in the past few years, due to the potential beneficial effect on photovoltaics^1^. Efficient CM has been demonstrated in colloidal quantum dots (QDs)^2^,^3^,^4^, nanorods^5^,^6^,^7^,^8^ and carbon nanotubes^9^. The work on QDs, in particular, has been extensive and the large majority of papers have focused on CM in colloidal dispersions of QDs. However, to be of use for photovoltaics, CM needs to be efficient in solid-state materials, rather than dispersions, and only a few papers have appeared on CM in films of QDs^10^,^11^,^12^,^13^. Moreover, CM should ultimately result in enhanced photocurrent, which means that the generated electrons and holes should be mobile. Here we use the term CM to refer to the generation of multiple charge carriers by a single photon. These carriers may reside in Coulomb-bound excitons or they may be free charges; only in the latter case do the charge carriers contribute to photoconductivity. To distinguish these two situations, we introduce the term Multiple Free Charge carrier Generation (MFCG), which requires the generation of multiple e–h pairs via CM followed by the dissociation of bound e–h pairs into free charge carriers.

A recent large step forward was made by Semonin *et al.*^14^ when they showed that CM is active in PbSe QD solar cells and can result in an external quantum efficiency exceeding 100%. This direct demonstration of enhanced photocurrent in QD solar cells demonstrates the occurrence of MFCG beyond any doubt. However, MFCG was only observed for certain QD solar cells in which the QDs were treated in part with 1,2-ethanedithiol ligands and in part with hydrazine. An explanation for this observation has not been given. A similar variation in the CM efficiency for various surface treatments of QDs was observed, but not explained, in an earlier paper by Beard *et al.*^11^.

Here we reveal the reason for the varying MFCG efficiency in QD films. We varied the mobility of charge carriers in QD films systematically over more than two orders of magnitude and determined the yield of charges in these films. We demonstrate a clear correlation between mobility and MFCG efficiency. These results are explained quantitatively by the competition between Auger recombination and the (mobility dependent) dissociation of multiple e–h pairs into the bulk of the QD film. For mobilities in excess of 1 cm^2^ V^−1^ s^−1^, all electrons and holes separate rapidly enough to escape Auger recombination and the MFCG efficiency saturates at a level that is very similar to the CM efficiency for colloidal QD dispersions. We further find that the threshold energy (the photon energy where CM sets in) is reduced to twice the band gap in QD films with short organic ligands, whereas the threshold energy is 2.8*E*_g_ for the same QDs in solution. The cause of this threshold reduction is not fully clear, but it is a promising observation for the exploitation of CM in solar cells, as a reduction in the threshold to 2*E*_g_ has been shown to be the major challenge for obtaining solar cell efficiencies in excess of the Shockley–Queisser limit^1^.

## Results

### Quantum-dot solids with varying ligand length

PbSe quantum dots of 6.0 nm diameter were synthesized following (ref. 15). During the synthesis, the QDs were capped with oleic acid, which acts as a barrier for electronic transport between QDs. We adopted the layer-by-layer (LbL) deposition procedure for making photoconductive QD films. During this procedure, the original lengthy ligands were replaced with shorter ligands of various lengths. The exchange ligands used were 1,2-ethanediamine (2DA), 1,3-propanediamine (3DA), 1,4-butanediamine (4DA), 1,6-hexanediamine (6DA) and 1,2-ethanedithiol (2DT). By varying the carbon chain length of these ligands, the mobility of electrons and holes is modified in a controlled manner^16^,^17^,^18^. Varying the anchor group of these ligands is also known to affect the mobility^18^. Details of the sample preparation procedures can be found in the Methods section.

**Figure 1:**
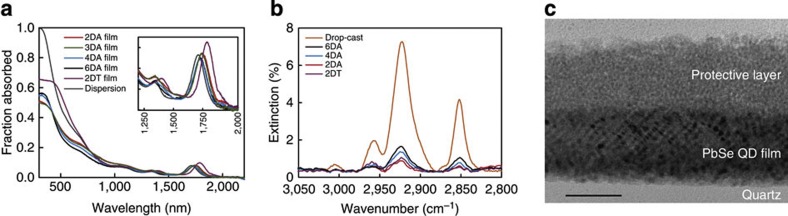
Characterization of the prepared QD films. (**a**) Absorptance spectra of the PbSe QD dispersion and PbSe QD LbL films. The inset shows a zoom-in around the band gap. (**b**) Fourier transform infrared spectra of a drop-cast PbSe film (with original long ligands) and films prepared with short replacing ligands. The extinction due to C–H stretch vibrations scales with the carbon chain length of the ligands. (**c**) Cross-sectional TEM image of a PbSe QD film with a protective CdSe QD layer on top. Scale bar, 50 nm.

Figure 1a depicts the absorptance spectra of the prepared PbSe QDs dispersed in tetrachloroethylene along with spectra of various QD films. The absorption measurements were performed in an integrating sphere to accurately correct for scattering and reflection. Compared with the QD dispersion, the first absorption peak in the spectra of the films exhibits a small red shift, and some broadening, as is common for PbSe QD films^16^,^19^. Figure 1b shows Fourier transform infrared spectra of the prepared LbL films along with a drop-cast film with the original oleic acid ligands normalized to the optical density of the first exciton peak. The reduction in the C–H stretch vibrations indicates the exchange of the long ligands by shorter ones in the LbL films. The extinction due to the C–H stretch vibrations increases with the length of the carbon chain of the exchange ligand, as expected. Figure 1c shows a cross-sectional transmission electron micrograph (TEM) of a QD film that was similar to the films studied below. This film consists of 10 layers of PbSe QDs with 2DA ligands on a quartz substrate covered with 10 layers of CdSe QDs with EDA ligands. The CdSe QD layer serves as a protective layer that prevents damage to the PbSe QD layer in the polishing of specimens for the cross-sectional TEM measurements and was not present in the other experiments described here. The individual PbSe QDs in the film can be seen in the micrograph. Taken together with the sharp excitonic features in the absorptance spectra (Fig. 1a), we conclude that the QDs remain distinct in the film and that quantum confinement is retained.

**Figure 2:**
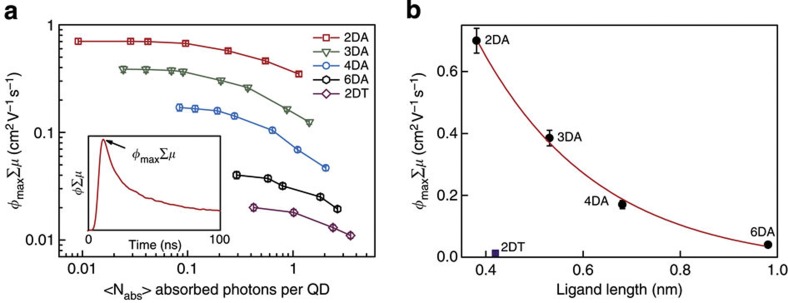
Photoconductivity of the QD films. (**a**) *φ*_max_Σ*μ* values as a function of the average number of absorbed photons per QD for QD films with various replacing ligands. The inset shows a typical photoconductance transient and the point that corresponds to *φ*_max_Σ*μ*.(**b**) *φ*_max_Σ*μ* values for alkyl diamine ligands with different carbon chain lengths. Note that 2DT shows a much lower *φ*_max_Σ*μ* value than 2DA. Error bars indicate the s.d.

The photoconductance of these films was measured using the time-resolved microwave conductance (TRMC) technique, which probes the ac photoconductance of the QD films without the use of contacting electrodes^20^,^21^. The QD films were photoexcited with nanosecond laser pulses of tunable wavelength, resulting in the generation of charge carriers. The accompanying increase in conductance is determined by measuring the absorption of microwave radiation. As shown in the inset of Fig. 2a, charge generation during the laser pulse results in an increase of the measured conductance, followed by a decrease as the charges become trapped or recombine.

Figure 2a shows the maximum value of the photoconductance transients as a function of excitation density, expressed as the average number of photons absorbed per QD, <*N*_abs_>. In these measurements, the photon energy was equal to the band gap energy of the QDs. The photoconductance is normalized to the number of absorbed photons and expressed as *φ*_max_Σ*μ*, where *φ*_max_ is the yield of mobile charge carriers per absorbed photon at the maximum of the photoconductance transient and Σ*μ* is the sum of the electron and hole mobility. *φ*_max_Σ*μ* decreases as the photoexcitation density increases due to higher-order recombination within the nanosecond laser pulse^22^,^23^,^24^. At low excitation density, no higher-order recombination takes place and the value of *φ*_max_Σ*μ* is independent of the photoexcitation density. The decay kinetics can be found in Supplementary Fig. S1.

Recently, we have shown that the yield of charge-carrier photogeneration is unity in PbSe QD films with 2DA ligands^22^. The 2DA-treated PbSe QD films investigated here were prepared identically to the films used in our earlier report and show very similar *φ*_max_Σ*μ* values, indicating that their charge generation yield is also unity. Figure 2b shows the plot of *φ*_max_Σ*μ* values as a function of ligand length, which was estimated using the semi-empirical method AM1 in Spartan'02. 2DA has an estimated length of 0.38 nm; the length of 2DT is 0.44 nm. For the longer alkyldiamines a nominal length of 0.125 nm of each C–C bond is assumed^16^. The data matches the distance dependence of tunnelling conductance as evident from the exponential fit to the data (red line in Fig. 2b). As the variation of *φ*_max_Σ*μ* for various diamine ligand lengths is well explained by the variation of Σ*μ* alone, we conclude that *φ*_max_ is unity for all films investigated here^18^. The 2DT film, although comparable in ligand length to 2DA, shows a much lower *φ*_max_Σ*μ*, in agreement with previous observations^18^.

### Multiple free charge-carrier generation

**Figure 3:**
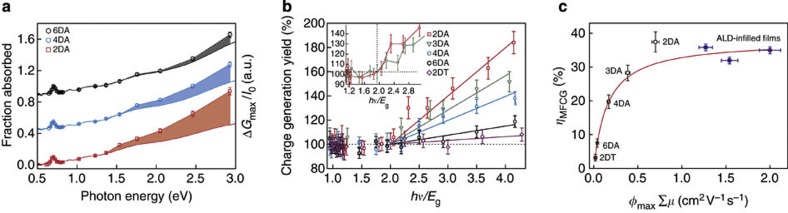
Multiple free charge-carrier generation in the QD films. (**a**) Left axis shows the fraction of absorbed photons for the QD films (solid lines). The right axis corresponds to the photoconductance normalized to the incident laser fluence (open data points). The shaded areas indicate MFCG. Data for the 4DA and 6DA films are offset for clarity. (**b**) Charge generation yield as a function of *hν*/*E*_g_ for all films. Straight lines are linear fits to the data assuming an energy threshold of 2*E*_g_. The inset shows a magnified view around 2*E*_g_ for the 2DA and 3DA films. (**c**) Multiple free charge-carrier generation efficiency plotted against *φ*_max_Σ*μ* values. Open squares are for films with organic ligands, solid blue circles are for ALD-infilled films from (ref. 29). The red line is the best fit to the data using equation 3. Error bars indicate the s.d.

To investigate MFCG, the films were excited with various photon energies *hν*, while ensuring for each measurement that the photoconductance was in the low excitation density limit. Figure 3a shows the photoconductance normalized by the incident photon fluence (Δ*G*_max_/*I*_0_, open symbols) for the 2DA, 4DA and 6DA films along with their absorptance spectra. The spectral shape of Δ*G*_max_/*I*_0_ coincides with the optical absorptance (see Supplementary Fig. S2 for a zoomed-in view) for photon energies ≤1.5 eV (~2*E*_g_). For these energies, the quantum yield for charge-carrier photogeneration is independent of photon energy and, as mentioned above, is ~100% (ref. 22). For photon energies >1.5 eV, Δ*G*_max_/*I*_0_ increases faster than the absorptance (shaded areas in Fig. 3a). This indicates that for photon energies above 2*E*_g_ the quantum yield exceeds 100% and MFCG occurs. MFCG is most pronounced for the film with 2DA ligands, but is also present for 4DA and 6DA films (blue and grey shaded areas in Fig. 3a, respectively). Similar traces obtained for all the films can be found in Supplementary Fig. S3.

In recent literature, a lot of emphasis has been put on the effect of photocharging on the apparent CM efficiency determined from ultrafast measurements^25^,^26^,^27^,^28^. In such measurements, the number of generated charges is extracted from the decay of transient absorption signals. Photocharging induces additional decay channels and artificially increases the extracted CM yield. The effect of photocharging, if present, on the current measurements is very different. First we note that the repetition rate of our TRMC experiments is very low (10 Hz), and we observe a full return to zero of the photoconductivity. This observation suggests that photocharging does not influence our measurements, as residual charges would contribute to photoconductivity. Second, we note that if photocharging influences our results it would be to reduce, rather than increase, the apparent MFCG efficiency. We determine the MFCG yield from the amplitude of the photoconductivity, which scales with the number of mobile charges generated by the laser pulse. In case of photocharging, this amplitude, and thus the MFCG yield, would decrease due to Auger recombination. The increase in photoconductivity at high photon energy, which we attribute to MFCG, cannot be induced by photocharging.

The charge generation quantum yield is obtained by dividing Δ*G*_max_/*I*_0_ by the fraction of absorbed photons and normalizing the quantum yield at the band gap to 100%. Figure 3b shows the charge generation yield versus the photon energy normalized to the band gap (that is, *hv*/*E*_g_). It is clear that there exist large variations in MFCG for the various exchange ligands: films with 2DT ligands show virtually no MFCG (purple diamonds in Fig. 3b), whereas the process is most efficient in films with 2DA ligands (red squares).

Another striking observation from Fig. 3b is that the charge generation yield starts to exceed 100% at a photon energy of ~2*E*_g_. This energy value, referred to as the threshold energy, has been reported to be ~2.8*E*_g_ for CM in colloidal dispersions of PbSe QDs^30^. A reduced CM threshold energy, to values of 2.33*E*_g_^6^ and 2.6*E*_g_^7^,^8^, was recently reported in one-dimensional PbSe nanorods. It is evident that the threshold is reduced even further in the QD films investigated here.

The reason for the threshold reduction is not clear. We speculate that it may be caused by changes in the band structure due to electronic coupling between QDs. If such changes result in asymmetric transitions, where all excess energy is stored in either the electron or the hole, this could lead to a reduction of the CM threshold to 2*E*_g_. It is also conceivable that trap states are involved in a trap-assisted impact ionization process. In such a process, an electron from an occupied trap state or a hole from an unoccupied trap state, may be ionized by hot carriers with an excess energy of less than the band gap. Alan *et al.*^31^ have calculated for Si QDs that sub band gap trap states may lead to a reduction of the CM threshold.

In the literature, the CM efficiency is defined as the linear increase in charge generation quantum yield per band gap multiple^30^,^32^. Here we define an analogous efficiency of MFCG, *η*_MFCG._ We fitted straight lines to the data keeping 2*E*_g_ as the energy threshold. The slope of the fits gives the MFCG efficiency that is plotted in Fig against the corresponding *φ*_max_Σ*μ* values. Open squares are for the films discussed so far. From the data, it is clear that the MFCG efficiency depends strongly on mobility. To test how general this observation is, we compare the films discussed here with QD films that were infilled with Al_2_O_3_ or Al_2_O_3_/ZnO using low-temperature ALD^33^, and for which we recently reported the MFCG efficiency^29^. TRMC measurements show that ALD-infilling results in mobilities of the order of 1–2 cm^2^ V^−1^ s^−1^, even higher than the highest mobilities obtained with organic ligands. We note that the onset of MFCG in these ALD-infilled films is not at 2.0*E*_g_, but ~2.7*E*_g_, that is, close to the onset found in colloidal QD dispersions^29^. The MFCG efficiencies for these ALD-infilled films are included in Fig. 3c, and are close to the MFCG efficiency found in the 2DA-treated films.

## Discussion

**Figure 4:**
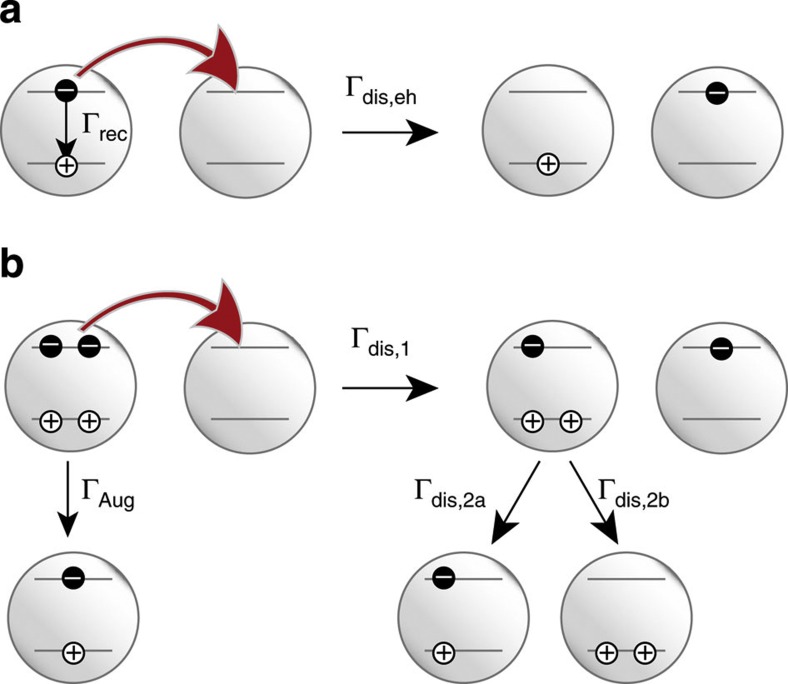
Dissociation of e–h pairs. Schematic of the dissociation of a single e–h pair (**a**) and multiple e–h pairs (**b**) into free charges in a QD film. The dissociation of single e–h pairs occurs in competition with recombination to the ground state, whereas the dissociation of multiple e–h pairs occurs in competition with Auger recombination. The dissociation of a double e–h pair involves multiple charge transfer steps. The first step is the rate-limiting step (see text). For simplicity, only QDs containing multiple charges are shown in the last step.

To understand the dependence of MFCG on the mobility, we consider the process of charge-carrier photogeneration. Figure 4a depicts the dissociation of single e–h pairs into free electrons and holes. The efficiency of dissociation of an e–h pair into free charges *η*_dis,eh_ depends on the competition of charge escape and recombination and is given by:


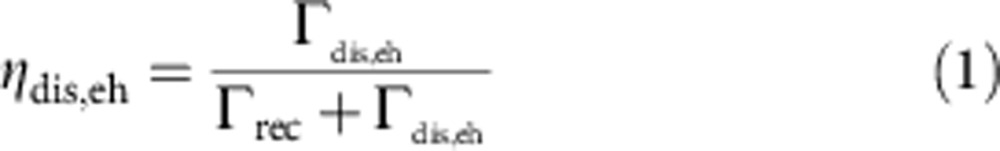


here Γ_rec_ is the e–h pair recombination rate and Γ_dis,eh_ is the e–h pair dissociation rate. We assume that the latter is given by the Miller–Abrahams rate^34^:


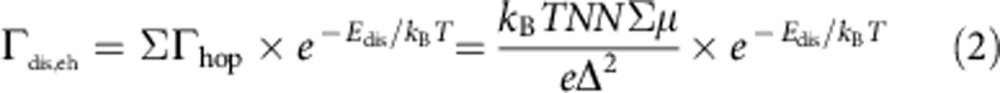


Δ is the hopping distance, *k*_B_ the Boltzmann constant, *T* the temperature and *e* the elementary charge. The dissociation energy *E*_dis_ reflects that charge separation requires the electron and hole to overcome their mutual Coulomb attraction. The dissociation rate depends on the sum ΣΓ_hop_ of the rates of electron and hole hopping between QDs, as the e–h pair can dissociate by either moving the electron or the hole. The hopping rate is derived from the mobility using the Einstein–Smoluchowski relation^35^ taking into account that the charges can hop to *NN* nearest neighbour sites. In a close-packed QD lattice, the number of nearest neighbours is 12. The e–h pair dissociation energy is estimated in Supplementary Note 1; a value of 58 meV is obtained, in line with the experimental observation of an electron–hole interaction energy of 80 meV in films of PbSe/CdSe core-shell QDs with a somewhat smaller PbSe core^36^. For 2DA ligands and a (non-radiative) exciton recombination rate Γ_rec_ of (15 ns)^−1^ (ref. 18), equations 1 and 2 predict a charge generation yield of 99.9%, in line with a previous report from our group^22^. Even for the 2DT ligands, with the lowest determined Σ*μ* value of 0.02 cm^2^ V^−1^ s^−1^, the estimated charge generation yield is 96%.

The situation changes when the photon energy exceeds the threshold for CM and multiple e–h pairs are generated per photon. These multiple e–h pairs may decay via Auger recombination, with a typical rate of (100 ps)^−1^ (ref. 37), that is, several orders of magnitude higher than the recombination rate of single e–h pairs. This situation is depicted in Fig. 4b. As the charge generation yields determined are below 200%, we restrict our analysis to dissociation of double e–h pairs, which involves the sequential dissociation into trions (path 1 in Fig. 4b), the further dissociation into single e–h pairs (path 2a) or doubly charged QDs (path 2b) and finally into free charge carriers. The rate-limiting step in this sequence is the dissociation of double e–h pairs into trions and free charges (path 1), as this path involves an increase in Coulomb energy whereas path 2a is Coulomb neutral. In addition, path 1 occurs in competition with Auger recombination of double e–h pairs, which is four times faster than the Auger recombination of a trion^37^, the competing channel for path 2a. Finally, path 2b is unlikely as it involves a significant increase in Coulomb energy. Hence, the escape yield of charges from double e–h pairs is well approximated by the yield of dissociation of double e–h pairs into trions (see Supplementary Note 2 and Supplementary Fig. S4 for further details).

The dissociation of double e–h pairs into trions results in two singly and oppositely charged QDs. The same is true for the dissociation of single e–h pairs and hence the dissociation energies of these two cases are similar. As there are twice as many charge carriers in a double e–h pair as in a single e–h pair, Γ_dis,1_=2Γ_dis,eh_. Finally, the efficiency of MFCG via CM is obtained as:


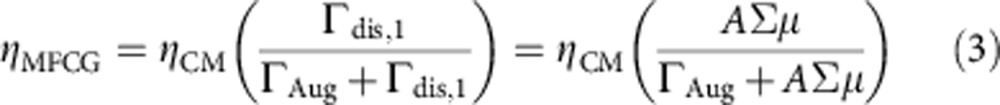


where Γ_Aug_ is the rate of Auger recombination and *A* is a proportionality constant that is given by:


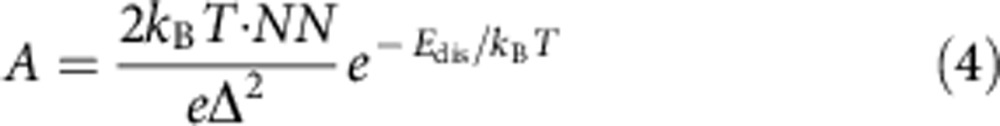


equation 3 is fitted to the data in Fig. 3c, resulting in the red solid line. The Auger recombination rate is taken as (100 ps)^−1^, corresponding to the value for biexciton recombination for PbSe QDs of similar size in solution^3^,^32^,^37^. An excellent fit is obtained with a CM efficiency of 37.9±1.5%, very similar to the value of 41% that is typically found in colloidal QD dispersions^30^.

From the fitted value of the parameter *A* an e–h pair dissociation energy of 3.1±0.2 *k*_B_*T* is extracted, in line with the 58 meV estimated above. According to the fit shown in Fig. 3c 85% of the double e–h pairs generated in the QD film with 2DA ligands escape Auger recombination. Increasing the sum of the electron and hole mobility enhances this value to 100% and yields a saturated MFCG efficiency, as evident from the fact that this efficiency is almost constant in the ALD-infilled QD films. Above a Σ*μ* value of 1 cm^2^ V^−1^ s^−1^, virtually all double e–h pairs dissociate into free charges and further optimization of the MFCG efficiency cannot be achieved via increasing the mobility.

The picture that emerges from this study is that the generation of multiple e–h pairs and their subsequent dissociation are independent processes. This is not surprising as the CM process itself occurs on a sub 100 fs timescale^3^, whereas dissociation of multiple e–h pairs and Auger recombination takes place on a picosecond to nanosecond timescale. The similarity between the CM efficiency obtained from these measurements and the CM efficiency determined in colloidal dispersions suggests that the CM process itself is not strongly affected by electronic coupling between QDs, or by ALD infilling. However, the observation that the threshold energy for CM is reduced does point to differences between QD films and QD dispersions that are not yet understood but that present a clear opportunity to further enhance the CM efficiency in QD films. Beard *et al.*^32^ have suggested that the CM efficiency and CM threshold are mutually connected. The observation made here that the threshold is reduced in films while the efficiency is similar as in colloidal dispersion suggests that this relation may not always hold.

In conclusion, our results show that CM occurs efficiently in PbSe QD films and that the generated multiple e–h pairs may be converted quantitatively to free charge carriers if the sum of electron and hole mobilities is high enough. This explains the previous difficulties in extracting multiple charges from colloidal quantum-dot films. The clear message from these results is that high charge-carrier mobilities are a prerequisite for exploiting CM in devices. In addition, we demonstrate that the energy threshold for CM is reduced to 2.0*E*_g_ in PbSe QD films with short organic ligands.

## Methods

### QD synthesis

Lead oxide weighing 0.66 g was dissolved in a mixture of 30 ml octadecene and 2.2 ml oleic acid. The solution was degassed at 100 °C under vacuum for 1 h. The solution was then heated to 180 °C in a Schlenk line under nitrogen atmosphere. The synthesis was carried out by injecting 10.8 ml of 1 M selenium (1 M Se in trioctylphosphine mixed with 84 μl of diphenylphosphine) swiftly to the lead precursor. The reaction mixture was kept at 160 °C for 2 min after which it was quickly cooled by a water bath. The QDs were precipitated with butanol and methanol and centrifuged at 5,000 r.p.m. for 5 min, redissolved in hexane and washed with butanol/methanol mixture once again. The QDs were then dispersed in tetrachloroethylene for absorption measurements and hexane for film preparation.

### LbL film preparation

To ensure good adhesion of the QDs to the quartz substrate, the substrate was first silanized with (3-aminopropyl)triethoxysilane. The PbSe QD films were prepared by layer-by-layer dip coating using a mechanical dip coater (DC Multi-8, Nima Technology) mounted inside a nitrogen glove box. The silanized quartz substrate was dipped alternately into a 10 μM solution of PbSe QDs in hexane for 60 s, a 1 M solution of the replacing ligands in methanol for 60 s and a rinsing solution of methanol for 30 s. The dipping procedure was repeated 15 times, resulting in homogenous thin films.

### Determination of the fraction of absorbed light

We used a Perkin-Elmer lambda 900 spectrophotometer equipped with an integrating sphere for the accurate determination of the fraction of absorbed light in the films. The absorption of the samples was recorded inside the integrating sphere, which corrects the reflection and scattering losses in the sample. The baseline spectra obtained from the quartz substrate, recorded in the integrating sphere under similar conditions is subtracted from the sample spectra to obtain the final absorption spectra.

### Cross-sectional TEM measurements

For the cross-sectional TEM measurements, 10 layers of PbSe QDs with 2DA ligands were dip-coated on a quartz substrate, on top of which another 10 layers of CdSe QDs were dip-coated. The CdSe QD layer serves as protective layer and helps to reduce the damage to the PbSe QD layer in the polishing of specimens for the cross-sectional TEM measurements. A glass substrate was then glued onto the film and a cross-section with a size of ~1 mm was cut from the film using a diamond saw. This was then mechanically polished to a thickness of 10 μm and loaded on to a copper TEM grid. The sample was then further thinned down to electron transparency using a Gatan PIPS 691 ion mill, using Argon. A Tecnai F20ST/STEM (200 kV) was used for imaging of the sample.

### Time-resolved microwave conductance

During the measurement, the samples were mounted in an X-band microwave cell (8.4 GHz). The sample could be illuminated via a grating in the copper end plate of the cell, which was covered and vacuum sealed with a quartz window. The other end of the cell was connected to a microwave source through a circulator detection scheme. The samples were photoexcited with 3 ns laser pulses from a tunable laser system (Opotek Vibrant 355 II). On photoexcitation, the change in microwave power reflected from the cell was measured. For small photo-induced changes in the real conductance of the sample, Δ*G*(*t*), and negligible change in imaginary conductance, the relative change in microwave power can be written as Δ*P*(*t*)/*P=−K*Δ*G*(*t*), where *K* is a sensitivity factor that has been determined previously. The photoconductance Δ*G*(*t*) can be expressed as Δ*G*(*t*)=*eβI*_0_*F*_a_Φ(*t*)Σ*μ*, where *e* is the elementary charge, *β* is the ratio between the broad and narrow inner dimensions of the waveguide, *I*_0_ is the photon fluence in the laser pulse, *F*_a_ is the fraction of light absorbed by the sample, Φ(*t*) is the number of mobile charge carriers at time *t* per absorbed photon and Σ*μ* is the sum of the electron and hole mobilities.

### Error analysis

The most prominent source of error in our TRMC measurements is in the determination of incident photon flux. This varied between 3–8% depending on the wavelength of excitation. Another source of error in the calculated photoconductivity is from the measurement of fraction of absorbed light, which varied between 0.1–3.0%, again dependent on the wavelength. These two errors are taken into account while plotting the error bars in Figs 2 and 3. The error bars in the MFCG efficiency plot (Fig. 3c) represent the s.d. from the linear fit to the data in Fig. 2b.

## Author contributions

C.S.S.S. prepared samples and performed experiments; S.T.C. performed experiments on the ALD-infilled samples; J.M.S. and T.J.S. advised on the measurements; Y.L. and M.L. provided ALD-infilled samples; A.J.H., S.K. and L.D.A.S. designed and supervised the project; A.J.H. conceived the experiments and analysis; A.J.H., C.S.S.S. and L.D.A.S. wrote the manuscript; all authors provided feedback on the manuscript.

## Additional information

**How to cite this article:** Sandeep, C. S. S. *et al.* High charge-carrier mobility enables exploitation of carrier multiplication in quantum-dot films. *Nat. Commun.* 4:2360 doi: 10.1038/ncomms3360 (2013).

## Supplementary Material

Supplementary Information Supplementary Figures S1-S4, Supplementary Notes 1-2 and Supplementary References

## References

[b1] Hanna M. C. & Nozik A. J. Solar conversion efficiency of photovoltaic and photoelectrolysis cells with carrier multiplication absorbers. J. Appl. Phys. 100, 074510 (2006).

[b2] Schaller R. D. & Klimov V. I. High efficiency carrier multiplication in PbSe nanocrystals: Implications for solar energy conversion. Phys. Rev. Lett. 92, 186601 (2004). 15169518 10.1103/PhysRevLett.92.186601

[b3] Ellingson R. J. *et al.* Highly efficient multiple exciton generation in colloidal PbSe and PbS quantum dots. Nano Lett. 5, 865–871 (2005). 15884885 10.1021/nl0502672

[b4] Trinh M. T. *et al.* In spite of recent doubts carrier multiplication does occur in PbSe nanocrystals. Nano Lett. 8, 1713–1718 (2008). 18489170 10.1021/nl0807225

[b5] Cunningham P. D. *et al.* Enhanced multiple exciton generation in quasi-one-dimensional semiconductors. Nano Lett. 11, 3476–3481 (2011). 21766838 10.1021/nl202014a

[b6] Cunningham P. D. *et al.* Correction to enhanced multiple exciton generation in quasi-one-dimensional semiconductors. Nano Lett. 13, 3003–3003 (2013). 10.1021/nl202014a 21766838

[b7] Padilha L. A. *et al.* Aspect ratio dependence of Auger recombination and carrier multiplication in PbSe nanorods. Nano Lett. 13, 1092–1099 (2013). 23360573 10.1021/nl304426y

[b8] Sandberg R. L. *et al.* Multiexciton dynamics in infrared-emitting colloidal nanostructures probed by a superconducting nanowire single-photon detector. ACS Nano 6, 9532–9540 (2012). 23020520 10.1021/nn3043226

[b9] Gabor N. M., Zhong Z. H., Bosnick K., Park J. & McEuen P. L. Extremely efficient multiple electron-hole pair generation in carbon nanotube photodiodes. Science 325, 1367–1371 (2009). 19745146 10.1126/science.1176112

[b10] Sukhovatkin V., Hinds S., Brzozowski L. & Sargent E. H. Colloidal quantum-dot photodetectors exploiting multiexciton generation. Science 324, 1542–1544 (2009). 19541992 10.1126/science.1173812

[b11] Beard M. C. *et al.* Variations in the quantum efficiency of multiple exciton generation for a series of chemically treated PbSe nanocrystal films. Nano Lett. 9, 836–845 (2009). 19170560 10.1021/nl803600v

[b12] Aerts M. *et al.* Free charges produced by carrier multiplication in strongly coupled PbSe quantum dot films. Nano Lett. 11, 4485–4489 (2011). 21939229 10.1021/nl202915p

[b13] Sambur J. B., Novet T. & Parkinson B. A. Multiple exciton collection in a sensitized photovoltaic system. Science 330, 63–66 (2010). 20929804 10.1126/science.1191462

[b14] Semonin O. E. *et al.* Peak external photocurrent quantum efficiency exceeding 100% via MEG in a quantum dot solar cell. Science 334, 1530–1533 (2011). 22174246 10.1126/science.1209845

[b15] Steckel J. S., Yen B. K. H., Oertel D. C. & Bawendi M. G. On the mechanism of lead chalcogenide nanocrystal formation. J. Am. Chem. Soc. 128, 13032–13033 (2006). 17017765 10.1021/ja062626g

[b16] Liu Y. *et al.* Dependence of carrier mobility on nanocrystal size and ligand length in PbSe nanocrystal solids. Nano Lett. 10, 1960–1969 (2010). 20405957 10.1021/nl101284k

[b17] Sun L. *et al.* Bright infrared quantum-dot light-emitting diodes through inter-dot spacing control. Nat. Nanotechnol. 7, 369–373 (2012). 22562037 10.1038/nnano.2012.63

[b18] Gao Y. *et al.* Photoconductivity of PbSe quantum-dot solids: dependence on ligand anchor group and length. ACS Nano 6, 9606–9614 (2012). 23078408 10.1021/nn3029716

[b19] Wolcott A. *et al.* Anomalously large polarization effect responsible for excitonic red shifts in PbSe quantum dot solids. J. Phys. Chem. Lett. 2, 795–800 (2011).

[b20] Haas M. P. D. & Warman J. M. Photon-induced molecular charge separation studied by nanosecond time-resolved microwave conductivity. Chem. Phys. 73, 35–53 (1982).

[b21] Kroeze J. E., Savenije T. J., Vermeulen M. J. W. & Warman J. M. Contactless determination of the photoconductivity action spectrum, exciton diffusion length, and charge separation efficiency in polythiophene-sensitized TiO_2_ bilayers. J. Phys. Chem. B 107, 7696–7705 (2003).

[b22] Talgorn E. *et al.* Unity quantum yield of photogenerated charges and band-like transport in quantum-dot solids. Nat. Nanotechnol. 6, 733–739 (2011). 21946709 10.1038/nnano.2011.159

[b23] Talgorn E. *et al.* Highly photoconductive cdse quantum-dot films: influence of capping molecules and film preparation procedure. J. Phys. Chem. C 114, 3441–3447 (2010).

[b24] Talgorn E. *et al.* Supercrystals of CdSe quantum dots with high charge mobility and efficient electron transfer to TiO_2_. ACS Nano 4, 1723–1731 (2010). 20184385 10.1021/nn901709a

[b25] Midgett A. G., Hillhouse H. W., Hughes B. K., Nozik A. J. & Beard M. C. Flowing versus static conditions for measuring multiple exciton generation in PbSe quantum dots. J. Phys. Chem. C 114, 17486–17500 (2010).

[b26] Padilha L. A. *et al.* Spectral dependence of nanocrystal photoionization probability: the role of hot-carrier transfer. ACS Nano 5, 5045–5055 (2011). 21591633 10.1021/nn201135k

[b27] McGuire J. A. *et al.* Spectroscopic signatures of photocharging due to hot-carrier transfer in solutions of semiconductor nanocrystals under low-intensity ultraviolet excitation. ACS Nano 4, 6087–6097 (2010). 20939512 10.1021/nn1016296

[b28] McGuire J. A., Sykora M., Joo J., Pietryga J. M. & Klimov V. I. Apparent versus true carrier multiplication yields in semiconductor nanocrystals. Nano Lett. 10, 2049–2057 (2010). 20459066 10.1021/nl100177c

[b29] ten Cate S. *et al.* Activating carrier multiplication in PbSe quantum dot solids by infilling with atomic layer deposition. J. Phys. Chem. Lett. 4, 1766–1770 (2013). 10.1021/jz4007492 26283107

[b30] Beard M. C., Luther J. M., Semonin O. E. & Nozik A. J. Third generation photovoltaics based on multiple exciton generation in quantum confined semiconductors. Acc. Chem. Res. 46, 1252–1260 (2013). 10.1021/ar3001958 23113604

[b31] Allan G. & Delerue C. Fast relaxation of hot carriers by impact ionization in semiconductor nanocrystals: role of defects. Phys. Rev. B 79, 195324 (2009).

[b32] Beard M. C. *et al.* Comparing multiple exciton generation in quantum dots to impact ionization in bulk semiconductors: implications for enhancement of solar energy conversion. Nano Lett. 10, 3019–3027 (2010). 20698615 10.1021/nl101490z

[b33] Liu Y. *et al.* Robust, functional nanocrystal solids by infilling with atomic layer deposition. Nano Lett. 11, 5349–5355 (2011). 22023409 10.1021/nl2028848

[b34] Miller A. & Abrahams E. Impurity conduction at low concentrations. Phys. Rev. 120, 745–755 (1960).

[b35] Gao Y. *et al.* Enhanced hot-carrier cooling and ultrafast spectral diffusion in strongly coupled pbse quantum-dot solids. Nano Lett. 11, 5471–5476 (2011). 22039918 10.1021/nl203235u

[b36] Swart I., Sun Z. X., Vanmaekelbergh D. & Liljeroth P. Hole-induced electron transport through core-shell quantum dots: a direct measurement of the electron-hole interaction. Nano Lett. 10, 1931–1935 (2010). 20392107 10.1021/nl100949a

[b37] Klimov V. I., McGuire J. A., Schaller R. D. & Rupasov V. I. Scaling of multiexciton lifetimes in semiconductor nanocrystals. Phys. Rev. B 77, 195324 (2008).

