# Supplementary material for: High charge-carrier mobility enables exploitation of carrier multiplication in quantum-dot films
Source: Nat Commun. 2013 Aug 23;4:2360. doi: 10.1038/ncomms3360 (PMC3759061; doi:10.1038/ncomms3360)
Supplement: Supplementary Information — Supplementary Figures S1-S4, Supplementary Notes 1-2 and Supplementary References [file ncomms3360-s1.pdf]

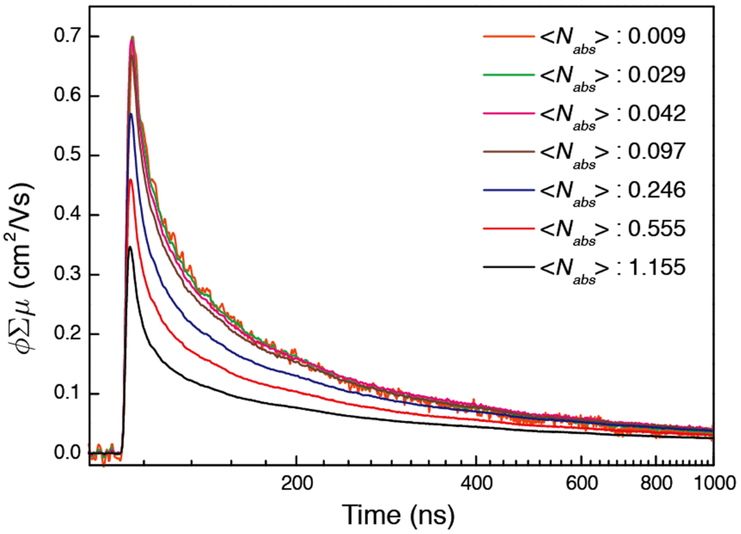

Supplementary Figure S1: Overlapping decay kinetics at low excitation densities, characteristic for first order recombination at these excitation densities.

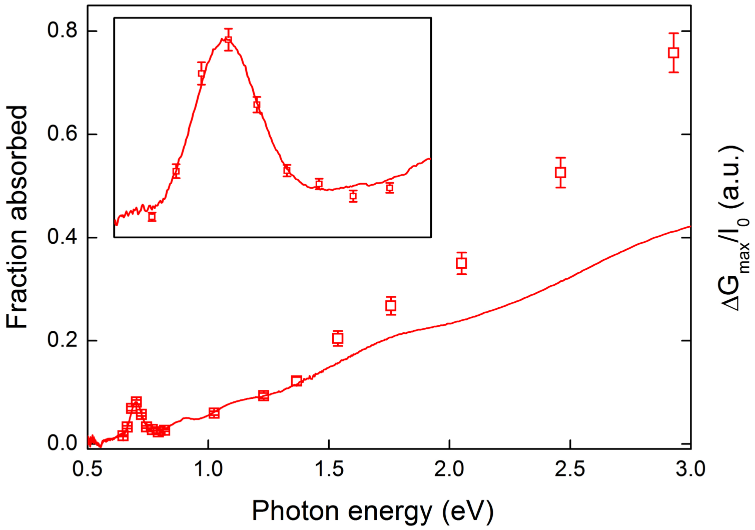

Supplementary Figure S2: Photoconductivity action spectrum for the 2DA sample. Inset shows a zoomed in view for energies around the bandgap.

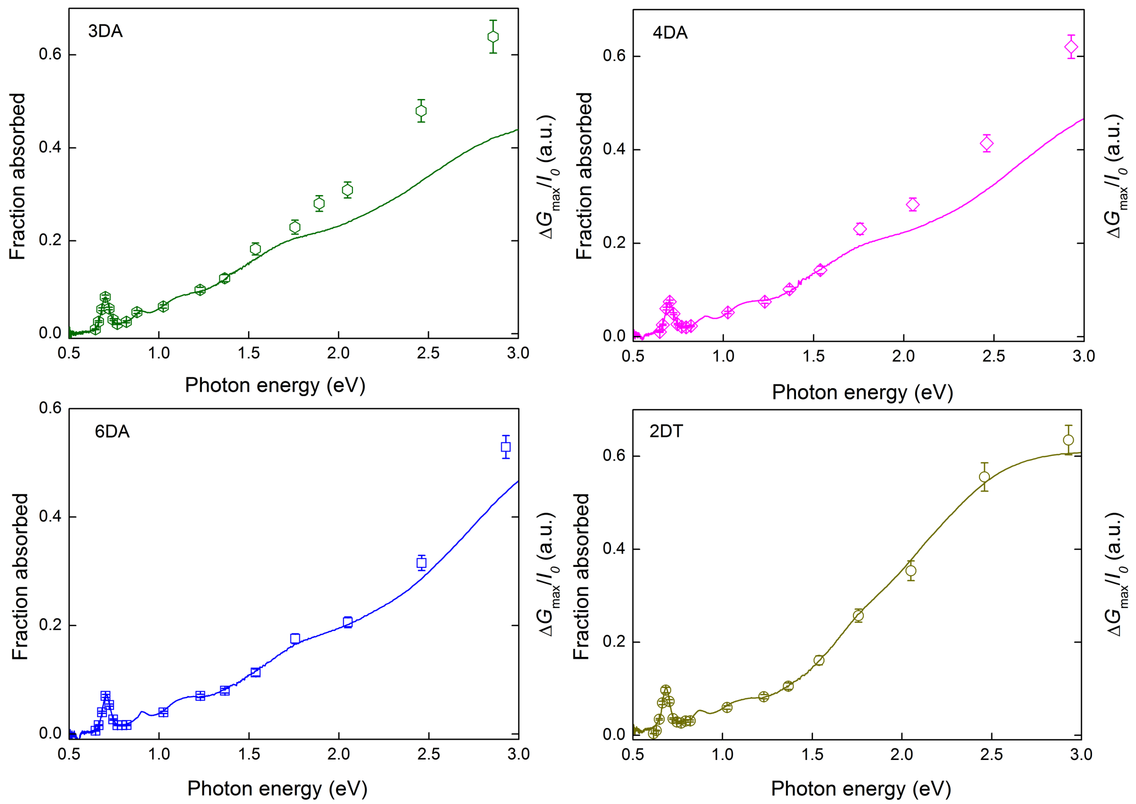

Supplementary Figure S3: Photoconductivity action spectra for all organic ligands. It can be clearly seen that for the 2DT film, there is hardly any multiple free charge carrier generation.

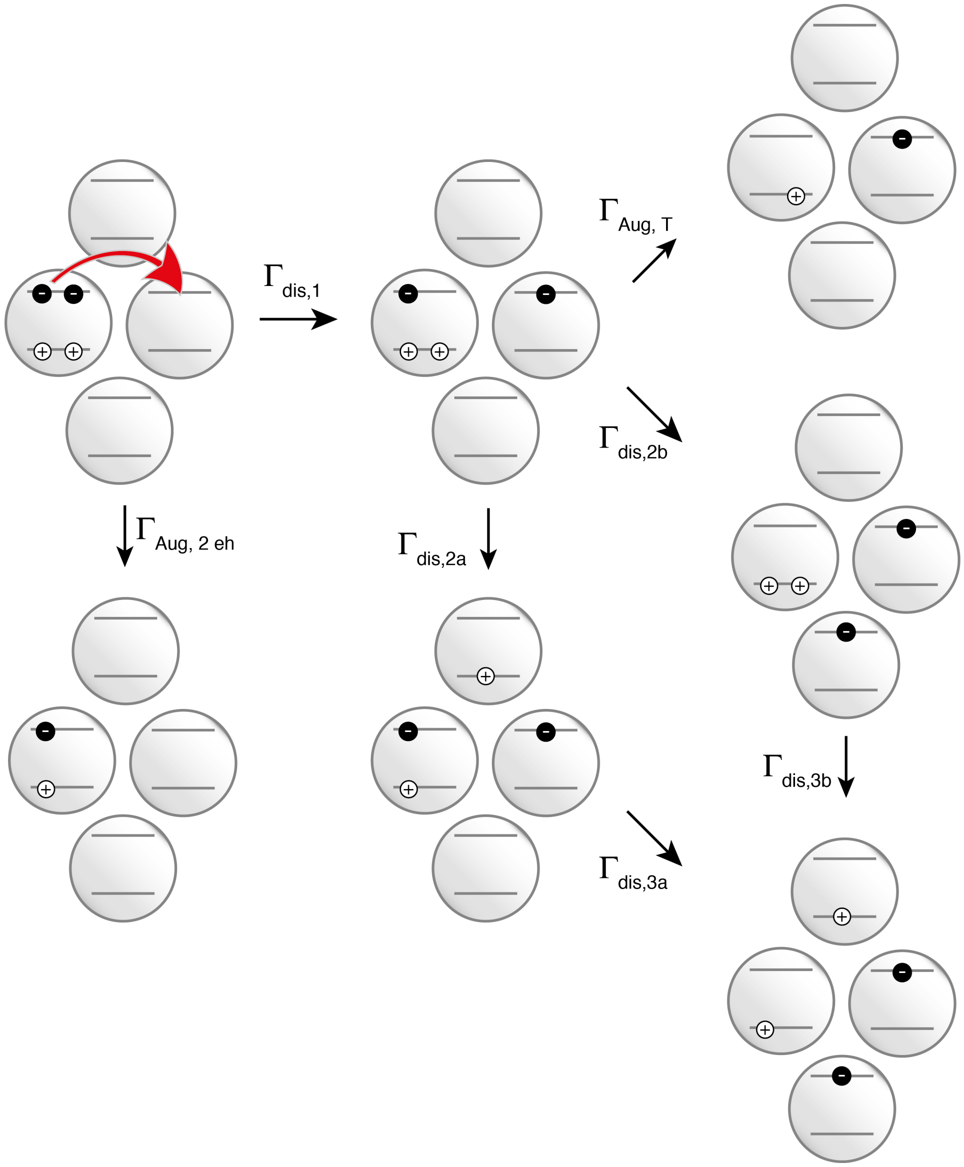

Supplementary Figure S4: Schematic of the dissociation of a double e-h pair into free electrons and holes in competition with Auger recombination.

Supplementary Note 1: Electron-hole pair dissociation energy

To estimate the dissociation energy of an e-h pair residing in one QD we first consider the energy E_∞ of a well-separated electron-hole pair where the electron and hole occupy different QDs and do not interact. With respect to the unexcited QD film the energy is:

$$E_\infty = E_{1\mathrm{e}} - E_{1\mathrm{h}} + E_{\mathrm{e}}^{\mathrm{pol}} + E_{\mathrm{h}}^{\mathrm{pol}} \qquad \text{(S1)}$$

Here $E_{1\mathrm{e}}$ and $E_{1\mathrm{h}}$ are the site energies of the electron and hole, respectively and $E_{\mathrm{e}}^{\mathrm{pol}}$ and $E_{\mathrm{h}}^{\mathrm{pol}}$ are the electron and hole self energies. When the electron and hole are brought together on a single QD the energy becomes:

$$E_{e-h} = E_{1e} - E_{1h} + E_e^{pol} + E_h^{pol} + E_{e,h}^{dir} + E_{e,h}^{pol} \qquad \text{(S2)}$$

$E_{\mathrm{e,h}}^{\mathrm{dir}}$ is the direct Coulomb interaction between electron and hole and $E_{\mathrm{e,h}}^{\mathrm{pol}}$ is the interaction of the electron with the polarization induced by the hole, and *vice versa*. The e-h pair dissociation energy $E_{dis,eh}$ is obtained as the difference between E_∞ and $E_{\mathrm{e\text{-}h}}$ and contains the direct Coulomb interaction $E_{\mathrm{e,h}}^{\mathrm{dir}}$ between electron and hole, as well as the cross-polarization energy $E_{\mathrm{e,h}}^{\mathrm{pol}}$. Expressions for these contributions have been derived by Delerue resulting in the following final expression for the dissociation energy:[38]

$$E_{\mathrm{dis,\,eh}} = \frac{1.79e^2}{4\pi\varepsilon_0\varepsilon_{\mathrm{in}}a} + \frac{e^2}{4\pi\varepsilon_0 a}\frac{\varepsilon_{\mathrm{in}} - \varepsilon_{\mathrm{out}}}{\varepsilon_{\mathrm{in}}\varepsilon_{\mathrm{out}}} \qquad \text{(S3)}$$

where ε_0 is the vacuum permittivity, a is the QD radius, $\varepsilon_{\mathrm{in}}$ is the dielectric constant of PbSe QDs, and $\varepsilon_{\mathrm{out}}$ is the dielectric constant outside of the QDs.

Equation (S3) shows that the dissociation energy and the dielectric constants are directly related. A complexity comes from the fact that the dielectric constant is frequency dependent. In bulk semiconductors, the exciton binding can be determined experimentally, and the proper value of the dielectric constant can be determined.[39] With the magnitude of the exciton binding energy the angular frequency of the electron and hole changes. If this frequency is higher than the frequency of the optical phonons the use of the optical dielectric constant is appropriate, if the frequency is well below the optical phonon frequencies, the static dielectric constant applies[39]. We assume that for QDs the use of the optical dielectric constant is always more appropriate. This results from the fact that electrons and holes always have a high kinetic energy as a result of quantum confinement and the associated frequency will be higher than the optical phonon frequencies[40]

$\varepsilon_{\mathrm{out}}$ is determined by the capping molecules, in this case 1,2-ethanediamine, and neighboring QDs. We consider it to be the effective dielectric constant of the film and obtain an estimate of its value by applying the Bruggeman effective medium theory:[41]

$$f\frac{\varepsilon_{\text{in}}-\langle\varepsilon\rangle}{\varepsilon_{\text{in}}+\kappa\langle\varepsilon\rangle}=(f-1)\frac{\varepsilon_{\text{m}}-\langle\varepsilon\rangle}{\varepsilon_{\text{m}}+\kappa\langle\varepsilon\rangle} \tag{S4}$$

where f is the fill factor of the QDs, $\langle\varepsilon\rangle$ is the effective dielectric function of the film, *i.e.* $\langle\varepsilon\rangle=\varepsilon_{\text{out}}$ and ε_{m} the dielectric function of the capping material. For QDs with a radius of 3.0 nm, a capping layer of 0.2 nm and a total packing density of 0.7, the fill factor is 0.58. With the optical dielectric constant of PbSe (23.9) and 2DA (2.11) this results in ε_{out} = 10.3 and an $E_{dis,eh}$ = 58 meV.

Supplementary Note 2: Escape yield from double e-h pairs

Note that we define a difference between yield, an overall fraction of charges indicated by the symbol ϕ, and efficiency, the slope of a charge yield vs. energy, indicated by the symbol η.

The overall yield of free charge carrier generation is determined by the initial CM efficiency η_{CM} and the escape yield of multiple e-h pairs from recombination. The latter process is shown schematically in supplementary Figure S4. It involves the dissociation of double e-h pairs into positive or negative trions ($\Gamma_{dis,1}$), the further dissociation of those trions into single e-h pairs ($\Gamma_{dis,2a}$) or doubly charged QDs ($\Gamma_{dis,2b}$) and finally into single charges per QD ($\Gamma_{dis,3a}$ and $\Gamma_{dis,3b}$). The overall efficiency of generation of free charge carriers η_{MFCG} is thus given by:

$$\begin{aligned} \eta_{MFCG} &= \eta_{CM}\phi_{esc} \\ &= \eta_{CM}\phi_{dis,2eh}\phi_{dis,T}\phi_{dis,eh} \end{aligned} \tag{S5}$$

with ϕ_{esc} the yield of escape from Auger recombination, $\phi_{dis,2eh}$ the yield of dissociation of a double e-h pair into a trion and a single charge, $\phi_{dis,T}$ the yield with which a trion decays into a single e-h pair and a free charge, and $\phi_{dis,eh}$ the yield of dissociation of a single e-h pair into free charges.

As explained in the main text the term $\phi_{dis,eh}$ is always near unity and can be dropped from eq. S5. The rate of dissociation of single e-h pairs depends on the hopping rate of electrons and holes and the dissociation energy of an e-h pair:

$$\begin{aligned} \Gamma_{\mathrm{dis,eh}} &= \Sigma\Gamma_{\mathrm{hop}} e^{-E_{\mathrm{dis}}/k_{\mathrm{B}}T} \\ &= \frac{k_{\mathrm{B}}T \cdot \Sigma\mu \cdot NN}{e\Delta^2} \end{aligned} \tag{S6}$$

Here $\Sigma\Gamma_{\mathrm{hop}}$ is the sum of the hopping rates for electrons and holes, E_{dis} is the dissociation energy of an e-h pair, NN is the number of nearest QD neighbors, $\Sigma\mu$ is the sum of the electron and hole mobility and Δ is the interparticle hopping $\Sigma\Gamma_{\mathrm{hop}}$ distance.

The yield of dissociation of a double e-h pair into a trion and a free charge is given by:

$$\begin{aligned} \phi_{dis,2eh} &= \frac{\Gamma_{dis,1}}{\Gamma_{dis,1} + \Gamma_{Aug,2eh}} \\ &\approx \frac{2\Gamma_{dis,eh}}{2\Gamma_{dis,eh} + \Gamma_{Aug,2eh}} \end{aligned} \tag{S7}$$

As there are twice as many charge carriers in a double e-h pair as in a single e-h pair, and the energy of dissociating the former into a trion is in first approximation identical to the energy required to dissociate a single e-h pair (in both cases the initial state is neutral and the final state involves two QDs with a single net charge), it is found that $\Gamma_{dis,1} \approx 2\Gamma_{dis,eh}$. However, since the

Auger recombination rate is ~150 times higher than the non-radiative recombination rate of single e-h pairs [42], the yield of double e-h pair dissociation can be much lower than the yield of single e-h pair dissociation.

The dissociation of trions can occur via two paths, 2a and 2b in supplementary Figure S4, and the overall yield depends on the sum of the rates of these paths:

$$\begin{aligned}\phi_{dis,T} &= \frac{\Gamma_{dis,2a} + \Gamma_{dis,2b}}{\Gamma_{dis,2a} + \Gamma_{dis,2b} + \Gamma_{Aug,T}} \\ &\approx \frac{10\Gamma_{dis,eh} + 0.005\Gamma_{dis,eh}}{10\Gamma_{dis,eh} + 0.005\Gamma_{dis,eh} + \Gamma_{Aug,2eh}/4}\end{aligned} \quad \text{(S8)}$$

Trion dissociation occurs in competition with Auger recombination of trions, which is four times slower than Auger recombination of double e-h pairs[4343]. Path 2a does not involve an increase in the Coulomb energy of the system, and hence the rate of this process is ~Exp($E_{\text{dis}}/k_B T$) times higher than the rate of single e-h pair dissociation. For E_{dis} = 58 meV, as determined above, this amounts to a factor 10. Path 2b involves an increase of the Coulomb energy of ~2E_{dis}. In addition, only a single carrier is involved in this step, *vs*. two carriers in the single e-h pair dissociation. Hence the overall rate of path 2b is ~200 times lower than $\Gamma_{\text{dis,eh}}$ and it can be disregarded.

Upon comparing eqns. S7 and S8, it is clear that the yield of dissociation of a double e-h pair into a trion and a free charge is limiting the overall escape yield. In the limit of low escape yield the trion dissociation yield is 20 times higher than the double e-h pair dissociation yield. Hence the escape yield of double e-h pairs is well-approximated by the yield of their decay into a trion and a free charge.

Supplementary References

38 Delerue, C. & Lannoo, M. *Nanostructures theory and modeling*. (Springer, 2004).

39 Knox. *Theory of excitons*. (Academic press, 1963).

40 Lippens, P. E. & Lannoo, M. Comparison between Calculated and Experimental Values of the Lowest Excited Electronic State of Small Cdse Crystallites. *Phys. Rev. B* **41**, 6079-6081 (1990).

41 Bruggeman, D. A. G. *Ann. Phys.* **24**, 636–664 (1935).

42 Gao, Y. *et al.* Photoconductivity of PbSe Quantum-Dot Solids: Dependence on Ligand Anchor Group and Length. *ACS Nano* **6**, 9606-9614 (2012).

43 Klimov, V. I., McGuire, J. A., Schaller, R. D. & Rupasov, V. I. Scaling of multiexciton lifetimes in semiconductor nanocrystals. *Phys. Rev. B* **77**, 195324 (2008).
